# Hospital Expenditure.—The Commissariat

**Published:** 1895-11-02

**Authors:** 


### The Institutional Workshop.

### HOSPITAL EXPENDITURE.—THE COMMISSARIAT.

### IV.—THE NURSING STAFF IN PROPORTION TO BEDS.

By far the largest element in a hospital, outside the patients, consists of the nursing staff. The proportion of nurses to other non-patients varies in the widest manner—from large hospitals where there may be twice as many nurses as other officials, to small institutions where the nurses amount to no more than one-third, there being often a great waste of subordinate labour. The interesting point with regard to the nurses is, not their proportion to the remainder of the household, but their number in proportion to the patients.

In the following table the number of patients is calculated who fall to the share of each nurse, including in the nursing staff the matron or lady superintendent, the sisters, nurses, and probationers, but not, of course, the ward-maids. The number of beds is noted in order that the element of size may be tested as far as it bears on economy of nursing. The fractional figure represents the number of patients remaining over to be shared among every ten nurses:—

| | Average of Patients to each Nurse. | Average of Occupied Beds. |
|---|---|---|
| **London.** | | |
| German | 8 | 104 |
| Seamen's | 5·6 | 214 |
| Great Northern Central | 4·8 | 68 |
| Westminster | 4·6 | 176 |
| St. Mary's | 3·7 | 251 |
| Middlesex | 3 | 215 |
| Charing Cross | 2·6 | 150 |
| Royal Free | 2·6 | 112 |
| St. George's | 2·5 | 323 |
| St. Thomas's | 2·5 | 365 |
| North-West London | 2·5 | 38 |
| King's | 2·3 | 185 |
| London | 2·3 | 656 |
| Guy's | 2·3 | 411 |
| St. Bartholomew's | 2·2 | 536 |
| University | 2·1 | 186 |
| Metropolitan | 1·7 | 47 |
| **Provincial, Scotch, and Irish.** | | |
| Hull Royal Infirmary | 5 | 160 |
| Newcastle-on-Tyne | 5 | 256 |
| Sheffield General Infirmary | 4·8 | 89 |
| Kilmarnock | 4·8 | 68 |
| York County | 4·7 | 103 |
| Leeds General | 4·6 | 355 |
| Wolverhampton | 4·4 | 145 |
| Edinburgh Royal Infirmary | 4·4 | 690 |
| Glasgow Royal Infirmary | 4·2 | 543 |
| Halifax | 4·1 | 92 |
| Glasgow West Infirmary | 4 | 388 |
| Dublin Meath | 4 | 89 |
| Birmingham Queen's | 3·7 | 113 |
| Bradford | 3·6 | 161 |
| Bristol General Infirmary | 3·6 | 202 |
| Birmingham General | 3·5 | 231 |
| Norfolk and Norwich | 3·5 | 134 |
| Sussex County | 3·2 | 134 |
| Dundee | 3·1 | 155 |
| Ayr County | 3 | 54 |
| Derbyshire Royal Infirmary | 2·9 | 87 |
| Aberdeen | 2·8 | 157 |
| Oxford | 2·7 | 105 |
| Cambridge | 1·4 | 56 |

It will be seen from the above figures that economy in the nursing staff cannot be said to have any relation to the size of the institution. St. Thomas's, with 365 occupied beds, and St. George's, with 323, are exactly on an equality as regards the proportion of their nursing staff, with the North-West London containing only 38. And in the provinces, Hull Royal Infirmary, with five beds to each nurse, accommodates pretty exactly the same number of patients as Bradford Infirmary, where the proportion to each nurse is 3·6, or Aberdeen, where it is 2·8.

On the whole, nurses in London have fewer patients on their hands than those in the provinces, or in Scotland or Ireland. In all probability this results from the strong competition for training in London hospitals, which induces these institutions to make room for as many probationers as possible. The probationer is not of much use in her first months of training, and cannot possibly replace skilled nurses; in fact, the work of those who have the new arrivals on their hands is probably far more arduous than in places where the staff is relatively far smaller but less teaching is undertaken.

The London Hospital is leading the way in a very interesting experiment which provides a two months' preliminary course of instruction for the probationer in a separate establishment before she is allowed to come into contact with the patient. If it were possible to make some such course obligatory on all probationers, the result would probably be to keep the nursing staff within limits, and to get through the day's work with half the worry. Three patients a-head is not a high average for the members of the nursing staff to cope with, taking into due consideration day and night work. Where the average falls below this too large a preponderance of the unskilled element may be suspected, and the work will in all probability be found to be pressing at times with exceptional severity on the skilled nurses. Further statistics relating to this point would be interesting and instructive.

The extent to which the size of the nursing staff influences the cost of provisioning the hospital would be very easy to show if nurses in all hospitals were fed alike. But it requires very little calculation to perceive that even a slight increase in the cost of each individual per week speedily overbalances in a large establishment the gain of a decrease in numbers. One hundred and fifty nurses, for instance, boarded at the rate of 6s. a week, will cost £260 a year less than 125 boarded at 8s. a week, and this difference per head in the cost is insignificant compared with those which actually exist. Still, certain points stand out. The first four London institutions, showing an average of over four patients to each nurse, are among those whose average for provisions is lowest, and any abnormal excess of the nursing staff is instantly reflected in the housekeeper's budget.

Note.—The Middlesex and Addenbrooke Hospitals were omitted from the table in the article on page 33, setting forth the comparative cost of provisions per occupied bed, because, owing to the closing of these hospitals during part of 1893, the figures for that year do not represent their usual rate of expenditure. It should be mentioned that the figures for provisions, quoted in the "Annual," for both Middlesex and University College Hospitals, do not, as will be subsequently shown, by any means represent the whole cost of provisions in these hospitals.

